# Towards successful coordination of electronic health record based-referrals: a qualitative analysis

**DOI:** 10.1186/1748-5908-6-84

**Published:** 2011-07-27

**Authors:** Sylvia J Hysong, Adol Esquivel, Dean F Sittig, Lindsey A Paul, Donna Espadas, Simran Singh, Hardeep Singh

**Affiliations:** 1 Houston VA Health Services Research & Development Center of Excellence, Michael E. DeBakey Veterans Affairs Medical Center, Houaron, Texas, USA; 2 Department of Medicine - Health Services Research Section, Baylor College of Medicine, Houston, Texas, USA; 3 St. Luke's Episcopal Health System, Houston, Texas, USA; 4 University of Texas School of Biomedical Informatics and the UT-Memorial Hermann Center for Healthcare Quality & Safety, Houston, Texas, USA; 5 School of Social Work, University of Texas at Austin, Austin, Texas, USA; 6 Louis Stokes Cleveland VA Medical Center, Cleveland, Ohio, USA

## Abstract

**Background:**

Successful subspecialty referrals require considerable coordination and interactive communication among the primary care provider (PCP), the subspecialist, and the patient, which may be challenging in the outpatient setting. Even when referrals are facilitated by electronic health records (EHRs) (*i.e*., e-referrals), lapses in patient follow-up might occur. Although compelling reasons exist why referral coordination *should *be improved, little is known about which elements of the complex referral coordination process should be targeted for improvement. Using Okhuysen & Bechky's coordination framework, this paper aims to understand the barriers, facilitators, and suggestions for improving communication and coordination of EHR-based referrals in an integrated healthcare system.

**Methods:**

We conducted a qualitative study to understand coordination breakdowns related to e-referrals in an integrated healthcare system and examined work-system factors that affect the timely receipt of subspecialty care. We conducted interviews with seven subject matter experts and six focus groups with a total of 30 PCPs and subspecialists at two tertiary care Department of Veterans Affairs (VA) medical centers. Using techniques from grounded theory and content analysis, we identified organizational themes that affected the referral process.

**Results:**

Four themes emerged: lack of an institutional referral policy, lack of standardization in certain referral procedures, ambiguity in roles and responsibilities, and inadequate resources to adapt and respond to referral requests effectively. Marked differences in PCPs' and subspecialists' communication styles and individual mental models of the referral processes likely precluded the development of a *shared *mental model to facilitate coordination and successful referral completion. Notably, very few barriers related to the EHR were reported.

**Conclusions:**

Despite facilitating information transfer between PCPs and subspecialists, e-referrals remain prone to coordination breakdowns. Clear referral policies, well-defined roles and responsibilities for key personnel, standardized procedures and communication protocols, and adequate human resources must be in place before implementing an EHR to facilitate referrals.

## Background

Successful referrals require considerable coordination and interactive communication among the primary care provider (PCP), the subspecialist, and the patient, which may be challenging in the outpatient setting [1-3]. Several studies at the interface of primary and subspecialty care [4-9] suggest poor referral coordination and communication as an important contributor to delays in care,[10,11] mainly due to inappropriate timing and detail of information [12] and lost paperwork. The use of information technology has significant potential to improve care coordination [13]. For instance, referrals may be more successful when transmitted through an integrated electronic health record (EHR; *i.e*., e-referrals), allowing the PCP and subspecialist to exchange information electronically, and both have immediate access to the entire patient record. However, in recent work we found failures in referral completion despite e-referrals;[14] about 6% of e-referrals lacked timely follow-up by subspecialists, whereas when subspecialists discontinued or deferred e-referrals and returned them to PCPs for additional actions, 7% were lost to follow-up [15]. Incomplete prerequisite workup and subspecialists' determination that the referral was not required were cited frequently as reasons for discontinuing e-referrals. This suggests a better understanding of referral coordination and communication may be needed to maximize the benefits of an EHR to the referrals process [16].

Despite recommendations that referral coordination should be improved, [1,3,17] Available: http://www.biomedcentral.com/1472-6963/9/62, [18] the healthcare literature sheds little light on which elements of coordination should be targeted. Although a recent measurement framework of coordinated care is a start,[19] it does not identify the specific tools (*e.g*., routines, plans, schedules) and processes healthcare providers use to collectively and effectively transition patient care from primary to secondary care setting and vice versa [20,21]. However, literature from business management may provide guidance on operationalizing many elements of effective coordination and shed additional light on this issue.

### Elements of coordination: an integrative framework

Okhuysen & Bechky [22] propose an integrative framework explaining the mechanisms of coordination and the integrating conditions necessary to achieve it effectively. According to this framework, five basic organizational arrangements (*i.e*., mechanisms) allow individuals to accomplish a collective performance, that is, to coordinate:

1) *Plans and rules*: "purposive elements of formal organizations" [22] (p . 473); for example, who is allowed to place a referral request?

2) *Objects and representations*: technologies, tools, and any device used to "create a common referent around which people interact, align their work, and create shared meaning" [22] (p. 474); for example, how to use a template to place a referral request.

3) *Roles*: expectations of specific individuals; for example, which provider is supposed to follow-up with the patient after he/she visits the subspecialist?

4) *Routines*: "repeated patterns of behaviour that are bound by rules and customs" [22] (p. 477); for example, when a test result is completed, the ordering provider is notified.

5) *Physical proximity among team members*: for example, where are the referring provider and the subspecialist located--in the same building, and/or affiliated with the same institution?

These five basic mechanisms operate in various ways (*e.g*., by facilitating direct information sharing, developing agreement, creating common perspectives) to allow teams to achieve three integrating conditions, that is, the means by which people collectively accomplish their interdependent tasks: (1) accountability (clarity over who is responsible for what), (2) predictability (knowing what tasks are involved and when they happen), and (3) common understanding (providing a shared perspective on the whole process and how individuals' work fits within the whole). How these mechanisms and integrating conditions manifest themselves in the referrals process is not well described in the literature. Using this framework as an analytic guide, our study aims to provide insight into these relationships by identifying barriers, facilitators, and perceived solutions for improving communication and coordination of EHR-based referrals in an integrated healthcare system.

## Method

### Design and setting

This work is part of a larger study examining work-system barriers, facilitators, and suggestions for improving EHR-based communication.

**Figure 1:**
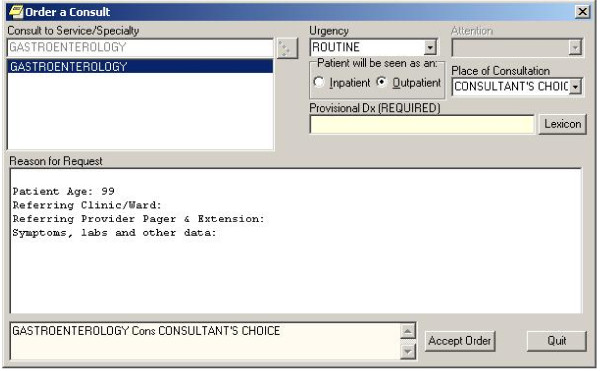
**Computerized Patient Records System (CPRS) referral order entry interface**. This figure presents an example of the interface where the primary care provider would place a request to refer a patient to a subspecialist. The provider can select the service needed, urgency, and must provide a provisional diagnosis; the provider then enters free text details of the reason for the request and any pertinent details about the patient's case.

Two large tertiary care Department of Veterans Affairs (VA) Medical Centers (Sites A and B) from different geographical areas served as study sites. The Computerized Patient Record System (CPRS) is the EHR used at all VA facilities (Figure 1); it integrates most aspects of clinical care and has comprehensive e-referral management functionality. Compared to nonintegrated systems, the VA is an ideal environment to study referral coordination because the universal use of the EHR by those who work in the same health system minimizes problems with information transmission [23].

We used subject matter expert (SME) interviews to document and understand the e-referral process workflow at four high-volume referral subspecialty clinics at Site A. These insights guided focus groups (FGs) to identify barriers, facilitators, and suggestions for improving the e-referral process at Sites A and B. Methods for this work have been described elsewhere [24] and are summarized here.

### Subject matter expert interviews

#### Participants and sampling frame

We purposefully sampled key informants, consisting of subspecialists, physician assistants, and administrative support staff, who were knowledgeable about referral processes within their subspecialties (n = 7). We interviewed one to two SMEs from each of four high-volume referral subspecialties (cardiology, neurology, pulmonary, and gastroenterology).

#### Procedure

We used a verbal protocol approach [25,26,26] with participants to elicit the process of using CPRS to receive, process, and complete or discontinue an e-referral. Responses were audio-recorded, captured in field notes, and used to create maps of the e-referral processes of each subspecialty and to inform the FGs.

#### Data analysis

Process maps were created for each subspecialty to capture the course of action for processing a referral from its reception to final outcome. Two independent coders (LAW and AE) analyzed the transcripts of each of the SME interviews to identify the various steps of all subspecialty referral processes. The coders used standard flowchart symbols to denote the process flow. The coders' versions of each map were validated by consensus to create final illustrations of each subspecialty. Comparison of the maps highlighted the large variability across specialty services; however, we identified activities shared across services based on their sequence within the overall referral process and their purpose. We used the final process maps as the foundation for creating the FG protocol and subsequent data analysis.

### Focus groups

#### Participants and sampling frame

We conducted six FGs with a total of 30 participants. We sampled purposefully to ensure a diversity of participants (*i.e*., PCPs who referred patients to the four selected subspecialties and subspecialists experienced in their respective referral procedures). Two FGs with PCPs (FGs 1 and 3) and two with subspecialists (FGs 2 and 4) were conducted at Site A. Subsequently, two FGs (PCPs and subspecialists, respectively) were conducted at Site B to triangulate findings and determine data saturation. FGs were conducted in a private conference room at each facility.

#### Procedure

An experienced facilitator conducted the FGs using a semistructured protocol. A primary note taker (with a background in qualitative methods) and a clinician (to provide clarification and context as needed) were included as part of the research team in each FG.

During the first two FGs, participants discussed barriers to and facilitators of the e-referral process and offered suggestions for improvement. Participants were encouraged to consider organizational-, task-, and human resource-related factors, in addition to technological issues. As part of the discussion, we presented the participants of FGs 3 and 4 with the themes frequently raised during FGs 1 and 2, checking for agreement and asking for additional detail where appropriate. To promote free and open discussions on sometimes opposing ideas from both groups, we did not reveal the source of the ideas. We also encouraged participants of subsequent FGs to volunteer their own barriers, facilitators, and suggestions for improvement. Discussions were digitally audiorecorded and transcribed.

#### Data analysis

The FGs (370 minutes total) yielded a total of 216 transcript pages. Using techniques adapted from grounded theory [27] and content analysis [28], two coders independently coded the transcripts using ATLAS.ti 5.2.17 (ATLAS.ti Scientific Software Development GmBH, Berlin, Germany) identifying perceived barriers, facilitators, and suggestions for improving the referral process. Based on this initial coding, the research team then iteratively developed, refined, and applied a coding taxonomy to capture the complexities inherent in the referral process. Any final discrepancies were resolved by consensus. This process yielded 120 individual codes categorized as perceived barriers, facilitators, and suggestions for improving the referral process using CPRS.

Next, the research team organized the code taxonomy into salient themes (also by consensus), considering each code's groundedness (*i.e*., how often it was mentioned by participants) and whether single or multiple providers mentioned the code. Finally, relationships among themes were identified by their potential influences in the overall referral process.

## Results

### Subject matter expert interviews

**Figure 2:**
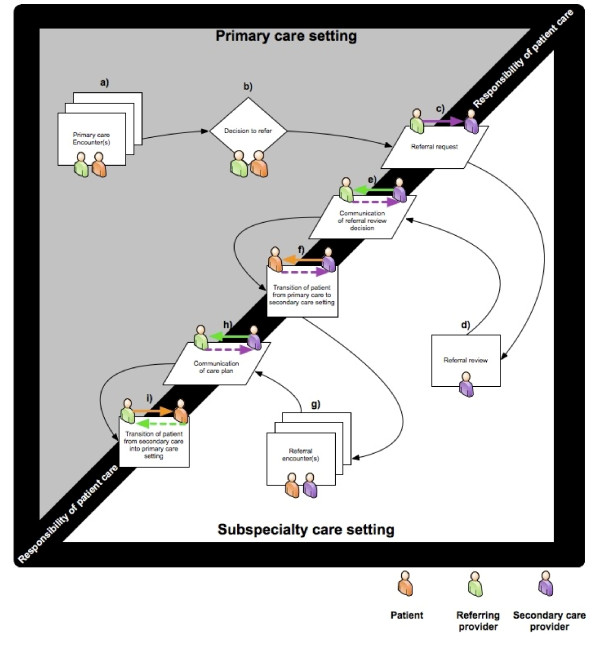
**Referral model based on subject matter expert interviews**. We identified three shared stages of the referral process based on the sequence and purpose of events and tasks: 1) submission of referral request by PCP; 2) referral review by the subspecialist; and 3) patient transition into subspecialty care. Referral requests are initiated using the EHR's order-entry interface (Figure 1). Upon receipt, subspecialists review requests to determine appropriateness, urgency and completeness, a process that could require additional information retrieval from the EHR. Subsequently, the referral is either: a) accepted and routed within the service to have an appointment scheduled; b) discontinued; or, c) deferred for further discussion with additional team members. Acceptance triggers a series of steps to coordinate patient transition into the subspecialty setting, including communication with patients to schedule appointments, followed by appointment reminders, an initial subspecialty encounter, and finally, communication of care plan back to the PCP through appropriate documentation of the referral encounter in the EHR.

Interview data were used to create detailed subspecialty-specific referral process maps that captured workflow, information transfer, and actions needed for processing referrals. We discussed these maps in several debriefing sessions and despite considerable variations across services, we identified a series of shared steps (Figure 2, steps a-i) in the referral processes based on the discussed sequences of events, goals, and tasks. These steps were consistent with previous work on developing a standardized model of the referrals process [29]. After one or more primary care encounters (step a), a decision to refer (step b) is made by the PCP. The PCP initiates the referral request (step c) using the EHR's order-entry interface, which permits the use of predesigned templates requiring variable amounts of information.

Upon receipt, subspecialists review the requests (step d) to determine appropriateness, urgency, and completeness, a process that sometimes requires detailed information retrieval from the EHR. Subsequently, the referral review decision is communicated (step e) to the PCP. Referrals can ultimately be (1) accepted and routed within the service to have an appointment scheduled, (2) discontinued, or (3) deferred for further discussion with additional team members.

If the referral is accepted, a series of steps are initiated that lead to coordinating the patient's transition into the subspecialty setting (step f), including communication with patients to schedule appointments, providing reminders, the referral encounter (step g) itself, the communication of the care plan (step h) to the PCP through appropriate EHR documentation, and finally, if appropriate, the coordination of the patient's transition back into the primary care setting (step i).

### Focus groups

The central emergent theme affecting coordination of e-referrals was the lack of an institutional referral policy. We also identified three additional themes that seem to result from the observed lack of policy: (1) no standardized practices for e-referrals, (2) ambiguous roles and responsibilities, and (3) inadequate resources to adapt and respond to incoming referral requests.

### Lack of policies and detailed instruction on e-referrals

Both PCPs and subspecialists perceived that lack of clear institutional policies for several critical steps of the outpatient referral process, such as rescheduling after no-shows and patient follow-up, was a barrier to successful referrals. For instance, they cited that the only two processes with an existing clear policy were mandatory referral requests for review within seven days of submission and scheduling of referrals within 30 days. However, instructions or procedures on how to successfully meet these requirements were lacking.

Subspecialists identified the large volume of referrals and difficulties reaching patients to schedule appointments as barriers to complying with the seven-day review/30-day scheduling policy. They acknowledged the policy to be well intended but lacked clear procedures to meet such high performance standards, which led to its poor implementation.

Well, it's reviewed within 7 and scheduled within 30. Um we have played around with that quite a deal, but it is impossible to get a patient scheduled within 30 days and it's not because of the triage process...but it's getting a hold of the patient... we contact every patient directly...we could send letters and we would get, we would be 100% within seven days, but then we would have no-show rates of 50% so... I think most of ours are reviewed within 12 days I think on average. --Subspecialist, FG 5

Subspecialists also commented about the need for clear policies and procedures for handling patients who do not keep their referral appointments as an important breakdown in the referral workflow.

I would like to see some institutional standards [about re-scheduling patients after patient no-shows], and I don't know that we have an institutional standard, but I think all of our patients and the providers would be much more aware if what was just spoken becomes the standard...If you miss two appointments in a row Jack, you're out. You may have a malignant condition. You could die, or whatever, or in this case that little weakness you had in your arm, that may be a sign Joe. You may be about to have a stroke, but if you had a standard then you can sell it. If you don't have a standard and it's different here and it's different there and it's different over there, then you really can't market. You can't advertise or promote it. --Subspecialist, FG 2

Notably, the most frequently raised suggestion for improving e-referrals was not technology upgrades, but the need to develop, disseminate, and implement a clear and comprehensive institutional referral policy.

### Lack of standardized practices for e-referrals

Lack of clear referral policies led to considerable variation in how different services reviewed and processed referrals. Process maps of the four services revealed considerable differences in what information was expected in the referral request, who reviewed the request, who made the final decision about the request, and what subsequent actions took place after a review decision.

### Referral content

In the referral request stage, PCPs and subspecialists disagreed on what they considered adequate content and ideal procedures for a referral request. PCPs perceived that some subspecialties had idiosyncratic referral requirements:

In my first year, I didn't know that a colonoscopy referral was different from Gastroenterology, so I put in a C-scope referral to Gastroenterology, and I didn't have the discontinued [notification] box checked off almost a year ago, because I didn't know. So obviously that was bad. So it's not because of an IQ problem. It's a system problem. If a GI [gastrointestinal] referral is placed, they need to forward it to C-scope. They need to take care of it. --PCP, FG 1

Conversely, subspecialists often cited a wide variation in the content of a referral request, some that they considered inappropriate or incomplete. They attributed this to PCPs' variable knowledge about proper referral techniques:

I think within [subspecialist's service] we also share that same problem. We get a lot of referrals - the patient has chest pain, and sometimes nothing is done... so I think we share that same philosophy. There is some education that needs to be done as a triage or pattern of how you get to this process. You don't just; well I'm having chest pain. Well have you assessed it? Is it musculoskeletal? --Subspecialist, FG 2

Participants offered multiple solutions to try to help minimize variation in the content of referral requests and develop a standardized way for PCPs and subspecialists to communicate. Suggestions included "information only" referrals, referral templates, and urgency flags. These proposed solutions sought to standardize how PCPs and subspecialists communicate, in order to develop a shared vision of what constitutes adequate e-referral content. However, PCPs and subspecialists disagreed on the potential effectiveness of these solutions.

### Information-only referrals

E-referrals did not allow PCPs to ask "curb-side" questions and obtain prompt responses before submitting formal requests. The only available options were either to call the subspecialist or schedule the patient for an appointment; thus, both PCPs and subspecialists suggested formalizing information-only referrals. In these requests, PCPs "ask" specific questions and subspecialists provide answers at their convenience without scheduling a future formal referral visit. Both sets of providers suggested this would reduce the volume of traditional visit-based referrals, decrease the amount of discontinued referrals (both by improving the quality of referral content and providing a formal venue for clinical questions), and ultimately improve relationships among PCPs and subspecialists.

I wouldn't mind having more [information-only referrals]... I don't necessarily want them [subspecialists] to see the patient. I want some guidance. --PCP, FG 3

The non-visit referrals are better... When people say what's the best approach for treating patients with heart failure? You know, and then you just give them a little blurb, okay, do this, do this and this, that's appreciated. Or just say does this patient need to be on anticoagulation? It's gold standard. Yeah, you do this... Those are specific little questions. I mean, that's when a nonvisit referral works and it is good. --Subspecialist, FG 4

### Referral templates

PCPs perceived that templates limited their ability to communicate clearly and caused frustration. They believed templates to be unilaterally designed by subspecialists for their own convenience. Furthermore, PCPs reported difficulties complying with prerequisites in some templates and often bypassed them altogether. They expressed concern that sometimes templates did not do justice to their clinical judgment, especially when they believed that the referral was required.

... they are trying to get me to put everything, copy and paste into [the template], copy and paste the MRI [magnetic resonance imaging], and copy and paste this and that, and it becomes redundant. I mean, a lot of times it even says on the template that if none of these [tests] are present and you put others and enter free text to give all the information, and if it is inappropriate, they'll discontinue it. --PCP, FG 3

In contrast, subspecialists strongly believed that creating more rigid templates (*i.e*., include more mandatory fields) could improve the quality and quantity of the information they receive.

*...you have to have the referral set up so that they [PCPs] will not be able to click past it unless they've done it ... templates where you have a number of questions that you have to answer and unless you answer them you can't go through, that's a sophisticated template...we need that*.

--Subspecialist, FG 2

### Urgency flags

Respondents reported that urgency flags on referral requests failed to influence the promptness of review. PCPs believed that subspecialists did not give it much consideration.

But there are other options like urgently or emergently or within a week, within a month, within a day, etc. I have no idea how various services treat our referrals and they all do it differently. I don't know whether or not if I put something to be seen within a week whether it really could happen or if it's just a dream that it could happen; and if it's a dream that it could happen then it shouldn't be there as one of the choices. --PCP, FG 5

### Ambiguous roles and responsibilities

Participants reported a clear disagreement over which provider (subspecialist vs. PCP) was responsible for specific tasks during various parts of the referral process, including information gathering, patient workup, and follow-up (both with the patient and the PCP).

### Information gathering

In the referral review stage, role ambiguity emerged as a greater barrier than the responsibility of gathering required information to make an assessment. Both PCPs and subspecialists believed that insufficient information in the e-referral request was a major reason for discontinuation; however, they had opposing views on what and how much information to include. Subspecialists emphasized that they made efforts to review more than what's included in the referral, but detailed EHR review for most patients was unrealistic due to the high referral volume. Conversely, PCPs argued for limiting the type and quantity of information they are expected to include because subspecialists had full EHR access.

If I was in the position where I'm going to discontinue what another physician has referred to me, I should access the electronic medical record and I should at least read the history. In some cases they just discontinue. Nobody reads the history. If we spend all the time to transcribe all the history [into the referral request], I think that is redundant because the electronic medical records make it easier for them [subspecialists] to access it and see exactly what I see. --PCP, FG 1

### Patient workup

Subspecialists perceived that PCPs placed many unnecessary referrals to shift the responsibility of appropriate workup to the subspecialists.

*Participant 11: But most of these referrals are placed for basically CYA [cover your ass]. It's a kind of shotgun, I know, but it's, it's not good medicine. It's the shotgun approach*.

*Participant 14: It's really overwhelming every single service*.

*Participant 11: But no one's, they're not thinking about it. They're just, they're already overwhelmed themselves*.

*Participant 14: Right, so they overwhelm everybody else*.

*Participant 11: So they're just, it's, they're just vomiting these referrals out*.

*Participant 14: It's a, it's a, a vicious, it's a vicious circle*.

--Specialists, FG 2

I wouldn't just put a referral in and have someone else do my thinking for me. But a lot of people, you know, will take the easy way and just [refer]. --Specialist, FG 2

In contrast, PCPs perceived that subspecialists discontinued referrals to avoid workload for which they were responsible.

They [the specialty service] said, oh you have to reschedule, you have to reorder this. I said why? The patient missed the appointment, why should I have to reorder the test? This is a total and complete waste of my time, and we got in a big wrangle about it 'cause I was like, why am I rescheduling something because he [the patient] missed the appointment? Reschedule it for me! He still needs it. I mean, why should I get involved? You know, and this is ridiculous. --PCP, FG 1

To help clarify areas of responsibility of information gathering and workup, some subspecialties implemented service agreements and e-referral guidelines for PCPs, including algorithms to help PCPs ensure their patients met certain referral criteria. However, PCPs exhibited mixed reactions to this solution; though well received by some, it was ignored, critiqued, or deemed pretentious by others. Conversely, PCPs strongly advocated for clear and extensive feedback from subspecialists when discontinuing their referrals.

### Follow-up with PCP: timely feedback and referral status updates

PCPs identified the lack of robust referral-tracking mechanisms as a major barrier. For instance, PCPs often felt uninformed when referrals were unresolved, discontinued, or even completed with no response from the subspecialist; they only found out when the patient returned to their clinics. Although some PCPs realized they might miss this communication among the volume of other electronic notifications received, others traced it back to subspecialists not providing timely feedback. In contrast, several subspecialists attributed this to PCPs voluntarily turning off their referral-related notifications.

The PCPs never find out unless they have their alerts turned on. Because they'll get a discontinued referral alert only if they have the alerts turned on. --Subspecialist, FG 2

### Patient follow-up

No clear VA policy existed to specify whether the PCP or subspecialist was responsible for patient follow-up about results of a test or procedure; consequently, PCPs and subspecialists disagreed over who was responsible for patient follow-up. This ambiguity was viewed as an important barrier to successfully processing referrals.

I don't like it when the specialist does the procedure, sends a letter to the patient saying you have tubulovillous adenoma, call your PCP for the information. [Even]if I don't understand tubovillous adenoma, [the patient] is going to call me. That is one whole call you made for me. That same PA [physician assistant] can call the patient and say hey, you have a polyp, that there's so and so risk, and you can follow up in five years. Why set up the PCP over there? --PCP, FG 1

Both provider types reported differences across services regarding who followed up with patients about tests ordered during or immediately after the referral encounter.

I think if the urologist is doing prostate biopsy, they should call them, or they should have a system. They all think PCP should do it... it's fine if the guy [patient] comes to me, but I'm not picking up the phone extra to call him in the middle of a unscheduled time to tell you hey, your urology report is so and so. I think that is the urologist, because he needs to tell him the plan. I'm not the one who's going to treat his cancer. --PCP, FG 1

### Resources to anticipate and respond to patient requests [22]

Adequacy of human resources appropriately skilled to schedule appointments, initiate reminders, or to reschedule patients after missed appointments was also cited as a barrier. Both parties agreed that current systems for direct, secure, and timely patient communication did not adequately address coordination of referrals.

People came to me and they said well, we'd like to have, you know, a central clerking system do this for you. The problem is--so the problem is thought--is that they don't have the knowledge base to know who needs x-rays, who doesn't need x-rays, which clinic to put them into. You know, you can try to give them that information, but they don't know the additional stuff that this person does. Unless they're trained, they wouldn't know that. And the problem is if you've got five or seven different clerks, you know, then they bounce, they change jobs every six months. I mean, we can't do it. --Subspecialist, FG 4

For example, some PCPs described situations where patients said they missed their appointment with the subspecialist only because they were never contacted. PCPs further commented on the difficulties patients sometimes faced, for example, when trying to reach subspecialty offices to schedule their own appointments. Conversely, several subspecialists discussed challenges when attempting to call patients or sending letters to outdated addresses.

...we mail letters to patients coming to our clinic, custom letters telling them about their appointment, how to prepare for a biopsy, how to prepare for a... but we have a parallel satellite mailing system because the letter-writing system does not work, or at least it does not work effectively. All our new patients get a personalized letter from our clerk, but it's not the VA letter. Our clerks mail a letter. Some of them may get two letters and we don't care. --Subspecialist, FG 2

Subspecialty services that implemented additional efforts to bolster patient-related communication (*e.g*., hiring additional staff or designating specific team members to contact patients and monitor transitions) perceived fewer difficulties in this context.

Actually, our clerk, she sends out a registered letter. If we don't get a hold of them [patients] within three days, she sends out a registered letter, and sometimes what we have to do is we have to move the appointment back because we haven't contacted the patient. We have this clerk that just does that. That's all she does. --Subspecialist, FG 2

Some of our patients don't call, and when they call, it's very difficult sometimes to get the call through and find the right person to talk to. --PCP, FG 1

## Discussion

We sought to understand coordination breakdowns that occur in an integrated healthcare system that used e-referrals; we also examined system factors that affect the timely receipt of subspecialty care. We elicited several barriers, facilitators, and suggestions for improving the coordination and timely receipt of subspecialty care. Salient themes included the need to (a) create concrete policies to clarify and standardize tasks and roles across subspecialties, (b) clarify ambiguity between PCPs and subspecialists on certain aspects of the referral process, and (c) ensure adequate resources for patient transition and follow-up. PCPs and subspecialists have quite different perspectives on improving e-referral processes, and bridging the divide will be an essential first step to improving coordination in this area. Qualitative data from studies such as ours can provide an appropriate and meaningful context to make e-referrals more successful.

Lack of clear and comprehensive policies that could provide detailed instruction to guide e-referrals was the central barrier. Both PCPs and subspecialists expected guidance from these policies to help clarify roles, responsibilities, and tasks, as well as to standardize key processes to achieve well-coordinated e-referrals. Clear policies and procedures are fundamental prerequisites to high performance, particularly for tasks involving high degrees of coordination;[30,31] this has been well documented in the industrial/organizational psychology and management literature [32,33]. In particular, Okhuysen & Bechky's [22]integrative framework of coordination details the conditions necessary to achieve effective coordination and puts our findings in context. According to this framework, effective coordination requires a clear and shared perspective of what is involved in the process (predictability), who is responsible for what part of the process (accountability), and how their share of the task fits into the whole (common understanding).

In the context of referrals, instructional aspects of policies act as the fundamental building blocks of common understanding, predictability, and accountability. Nevertheless, as Okhuysen & Bechky [22] suggest, policies by themselves are not sufficient to improve coordination and, in this case, successful referrals. Shared mental models (*i.e*., a common understanding of the goals, work involved, and roles of each team member in accomplishing those goals) are critical links between policy and the integrating conditions Okhuysen & Bechky propose [34-36]. Teams with strong shared mental models of the tasks and interactions tend to plan and coordinate better [37] and, ultimately, perform better than teams without a shared mental model [36,38]. In healthcare, similar instances have been documented where primary care clinic members sharing mental models of clinical practice guidelines were able to implement established guidelines more effectively [35].

In our research, we identified several barriers that, if addressed, would help improve accountability, predictability, and common understanding beyond what is accomplished by policy alone. For example, we found very distinct mental models about referrals, particularly with respect to roles, responsibilities, and communication of information throughout the referral process (accountability). We also found vast differences across services in how referrals are processed (predictability) and in how services follow up with providers and patients, attributable in part to the lack of policies, procedures, and communication protocols. Although our data could not confirm this, we believe the aforementioned differences may explain some of the varied opinions observed between PCPs and subspecialists about process improvement. Identifying the differences in the source of subspecialist mental models about the various aspects of referral coordination can be particularly helpful in achieving consensus between the two stakeholders. While our study does not provide all the needed answers at this stage, it does highlight the importance of the differences and information gaps. We believe this is an important area for future work in implementation science.

**Figure 3:**
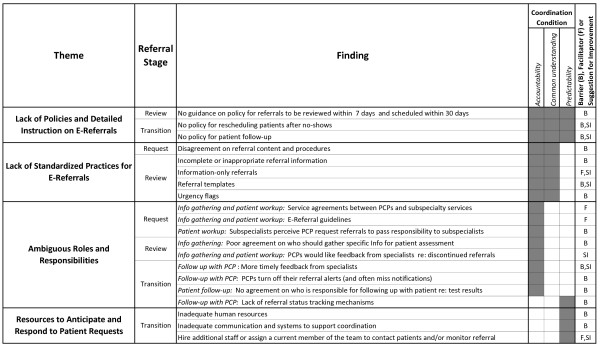
**Study findings in the context of the referral model stages and Okhuysen & Bechky's integrative coordination framework**.

Figure 3) presents our findings as they relate to the three main stages of the referral process (request, review, and transition to secondary care), in the context of Okhuysen & Bechky's framework. There were multiple barriers, facilitators, and suggestions for improvement within each theme, which manifested themselves most at specific stages of the referral process. For example, most findings about the lack of standardization related to the review stage and primarily constituted barriers regarding *objects/representations *and *routines *that hindered accountability and common understanding. Notably, the lack of policy (accomplished exclusively through *plans and rules*) hindered all three coordination conditions, which we interpret as evidence of its fundamental and central role in the referral process. In addition, the table also shows that barriers, facilitators, and suggestions for improvement existed in similar measure across all types of coordination mechanisms (except for *physical proximity*, which did not emerge at all in these data). Additionally, accountability was the integrating condition needing the most attention at these facilities to improve their referral process. This is consistent with the nature of referral work, which involves a transition of responsibility for care of a patient among multiple parties and requires clear accountability but relies on all five mechanisms of the Okhuysen & Bechky coordination framework for success.

The most notable finding, however, was that most barriers to successful e-referrals at these facilities were *not *due to difficulties with the EHR technology but rather basic issues of coordination and communication: ensuring everyone involved in the referral understood who needed to do or communicate and to whom and how each party's individual contributions affected the referral process as a whole [39]. Consistent with Venkatesh's Technology Acceptance Model, which proposes perceived usefulness and perceived ease of use as the primary drivers of technology acceptance,[40] participants offered specific technology-based solutions to some barriers, in an attempt to make the EHR more useful and easier to use and facilitate referrals. Some of these solutions, such as the use of templates and information-only referrals, have been implemented successfully in other systems [41] to address the lack of standardization in referral processes across services. Nevertheless, in both cases, the underlying problem addressed was not technological but, rather, one of coordination. Thus, by focusing on clarifying roles, standardizing procedures and communication of referral information, and implementing appropriate human resources, the referral process is more likely to result in timely and effective care, whether aided by an EHR or not.

### Improving referral coordination

The FG participants proposed solutions for some of the barriers raised, mostly focusing on the need for more specific policy (see Figure 3). Okhuysen & Bechky's framework provides additional guidance for addressing the other barriers raised during the FGs. For example, the two barriers in the request stage without proposed solutions (disagreement on referral content/procedures, subspecialists' perceptions that PCPs request referrals to pass responsibility to subspecialists) highlight the different perceptions of PCPs and subspecialists related to the content and process of referral requests. According to Okhuysen & Bechky, *roles *and *routines *help develop agreement and create a common perspective, thus promoting common understanding and subsequently facilitating coordination. Applying the framework to referrals, clear request procedures and agreement on what is considered appropriate content and prerequisite workup could resolve some of their differences; this would facilitate referral review and lead to fewer incomplete referrals, disagreements, and delays of care.

In the referral review stage, the problem of incomplete information continues, often resulting in the specialist referring to multiple locations in the EHR before being able to form a complete clinical picture, thus delaying care. According to the framework, *objects and representations*, such as automated summaries of the patient's current clinical condition, could facilitate direct information sharing and improve the common understanding between PCPs and subspecialists about the patient's situation. For example, research currently underway seeks to develop computer algorithms to aggregate, organize, and reduce a patient's computer-accessible clinical data and create a succinct summary of their past medical history [42]. Such automatically generated clinical summaries, combined with condition-specific referral initiation templates, could greatly improve the standardization and completeness of provider-to-provider communication.

In the transition stage, the common element underlying the reported barriers is the need for structure and resources that "make the progress of the task obvious,"[22] (p. 475) such as tracking systems, communication systems, and proper use of notifications. This is consistent with Okhuysen & Bechky's concept of "scaffolding" (adding structure to an object or representation as a reminder of remaining tasks and responsible parties), which is how *objects and representations *provide accountability and predictability.

### Limitations

The data for this study came only from VA sites using the same EHR, which may limit transferability to other contexts. However, our findings might be applicable to some extent beyond the VA for two reasons. First, the VA is comparable in many respects to large-staff model managed care organizations, such as Kaiser Permanente or Puget Sound Group Health [43], that refer to their own specialists and have electronic medical records. Second, with impending widespread adoption of integrated EHRs, formation of health information exchanges, [44] and accountable care organizations (ACOs),[45] information sharing between PCPs and specialists is only likely to grow and become more like other "integrated" health systems. Coordination for e-referrals in any system might be improved with clear policies, standardized procedures, and clarity of individual roles and contributions to the referral process that lead to stronger shared mental models.

## Conclusions

Whether aided by an EHR or not, referrals are fundamentally an exercise in coordination. Although an EHR is a powerful tool to help providers gather, organize, and transmit information, it cannot facilitate successful referrals in the absence of the basic fundamentals of coordination: (a) role clarity between PCPs and subspecialists, (b) standardization of referral-processing practices across specialties, and (c) adequate resources for patient transition and follow-up with both the patient and the PCP. Future work should clarify e-referral policies that (1) delineate roles and responsibilities for both primary care and subspecialty services and (2) standardize key referral requirements and procedures developed by all relevant stakeholders. These steps will build the shared understanding required for effective communication and coordination and foster effective behaviors needed to ensure referral success. Fundamental principles of coordination must be in place in order for EHRs to make a meaningful contribution to improving referral outcomes.

## Competing interests

The authors declare that they have no competing interests.

## Authors' contributions

SJH was responsible for the design of the qualitative methods, facilitated the focus groups, designed the analytic strategy, and had principal responsibility for writing the manuscript. AE led the execution of qualitative analyses and process map development and wrote portions of this manuscript. DFS aided in the later portions of the qualitative analysis and edited portions of this manuscript. LAW conducted the qualitative coding and analyses, aided in the conduct of the focus group, and materially edited the manuscript. DE conducted the subject matter expert interviews and coordinated the focus groups at Site A. SS coordinated and conducted the focus groups at Site B. HS is the principal investigator of the project, was responsible for the conceptual development of the research question, and materially edited the manuscript. All authors read and approved the final manuscript.
